# Development and characterization of a copolymeric micelle containing soluble and insoluble model drugs

**DOI:** 10.1371/journal.pone.0286251

**Published:** 2023-05-25

**Authors:** Farhad Mohammadi, Alireza Moradi, Fatemeh Tavakoli, Samaneh Rahmati, Rashin Giti, Vahid Ramezani

**Affiliations:** 1 Department of Pharmaceutics, Faculty of Pharmacy, Shahid Sadoughi University of Medical Sciences, Yazd, Yazd, Iran; 2 Department of Medicinal Chemistry, Faculty of Pharmacy, Shahid Sadoughi University of Medical Sciences, Yazd, Yazd, Iran; 3 Department of Pharmacology and Toxicology, Faculty of Pharmacy and Pharmaceutical Research Center, Shahid Sadoughi University of Medical Sciences, Yazd, Yazd, Iran; 4 Department of Prosthodontics, School of Dentistry, Shiraz University of Medical Sciences, Shiraz, Fars, Iran; Monash University Malaysia, MALAYSIA

## Abstract

**Objectives:**

Micelles are nano-sized particles with a core-shell structure that are made by natural or synthetic polymers or copolymers. The aim of this study was to develop and characterize a copolymeric micelle using two polymers loaded with hydrophilic and lipophilic drugs.

**Methods:**

Poly(ethylene glycol) and poly(ε-caprolactone) (PEG-PCL) were used to form a copolymeric micelle which was further loaded with either moxifloxacin or clarithromycin as hydrophilic and lipophilic drug samples, respectively. Characterization tests were done including fourier transform-infrared (FT-IR) spectroscopy, proton nuclear magnetic resonance (^1^H NMR) spectroscopy, encapsulation efficiency, particle size, zeta potential, polydispersity index, transmission electron microscopy, and *in-vitro* release test.

**Results:**

The construction of the copolymer was confirmed by the results of FT-IR and ^1^H NMR spectroscopy tests. The encapsulation efficiency test exhibited that loading was about 50% for twelve formulations. Particle size, zeta potential, polydispersity index, and transmission electron microscopy confirmed the formation of monodispersed, uniform, and nano-sized micelles with a few negative charges. The kinetic model of release was fitted to the Higuchi model.

**Conclusions:**

Polymeric micelles consisting of PEG-PCL copolymer were loaded with adequate concentrations of hydrophilic (moxifloxacin) and lipophilic (clarithromycin) model drugs, with a mean particle size under 300 nm. Therefore, copolymeric micelles can be used as a suitable drug delivery system for mucous membranes and skin.

## 1. Introduction

Polymeric micelles are a type of self-assembled nano-carrier that has recently received significant attention in drug delivery due to their low toxicity, improved bioavailability [1], targeted drug delivery, controlled drug release, high drug loading capacity, and enhanced drug solubility. The hydrophilic and hydrophobic blocks of copolymeric micelles form a core-shell nanostructure in aqueous solutions [2–4]. Their amphiphilic properties and capability to form micelles, along with their ability to load hydrophilic and lipophilic drugs, make them suitable for delivering various compounds such as anticancer drugs, proteins, nucleic acids, and contrast/imaging agents [5], as well as antibiotics to overcome antibiotic resistance and enhance therapeutic efficacy [6, 7]. In addition to these advantages, they can be smart and responsive systems to environmental stimulants such as pH, electric and magnetic fields, and ultraviolet waves [8].

Poly(ethylene glycol)-poly(ε-caprolactone) (PEG-PCL) is an FDA-approved biodegradable amphiphilic block copolymer, which has a low critical micelle concentration in aqueous solution (0.014 mg/ml), making it a commonly used for drug delivery [2, 9]. The hydrophilic segment of PEG is a biocompatible and targetable polymer that reduces clearance by the reticuloendothelial system and prolongs circulation time in the blood [10, 11]. On the other hand, the PCL block is a hydrophobic polymer that can serve as the inner core of micelles. It is biocompatible, non-toxic, biodegradable, and ideal for long-lasting drug delivery due to its slow degradation rate [12, 13].

Various drugs, both soluble and insoluble, can be loaded into polymeric micelles. Moxifloxacin (MFX) is a broad-spectrum fluoroquinolone antibiotic that inhibits a range of gram-negative and gram-positive organisms, anaerobes, and atypical bacteria [14]. Based on the Biopharmaceutics Classification System, it belongs to class I with high solubility and high permeability [15]. Clarithromycin (CLM), a macrolide antibiotic, has activity against gram-negative and gram-positive pathogens, atypical organisms, and some anaerobes [16]. Clarithromycin is practically insoluble in water and is classified as class II due to its poor solubility and high permeability [17, 18].

The use of PEG-PCL copolymer micelles as a carrier for loading hydrophilic and hydrophobic drugs has not been previously investigated. Therefore, in this study, the amphiphilic block copolymer (PEG-PCL) was synthesized and moxifloxacin as the hydrophilic and clarithromycin as the hydrophobic drugs were loaded separately on the micelles and their properties were characterized. The findings can be useful for loading both drugs simultaneously in a single dosage form.

## 2. Materials and methods

### 2.1. Materials

PCL, PEG (MW = 4000), 4-nitrophenyl chloroformate (NPC), CLM, MFX, pyridine, polyvinyl alcohol (PVA), Tween 80, methanol, and dichloromethane (all from Merck; Massachusetts, USA). Moxifloxacin and clarithromycin (Tehran Chemie; Tehran, Iran). All reagents and solvents were analytical grades.

### 2.2. Methods

#### 2.2.1. High-performance liquid chromatography (HPLC)

Standard serial dilutions of MFX and CLM in phosphate buffer saline were prepared, and 20 μL of each dilution was injected into an HPLC (Young Lin 9100; South Korea) three consecutive times on three different days. A C18 column (150 × 4.6 mm) was used and the mobile phase for CLM and MFX consisted of methanol: monobasic phosphate buffer (13:7) and methanol: buffer (28:72), respectively. The flow rate was set to 1 ml/min and the maximum ultraviolet absorption was read for CLM (210 nm) and MFX (270 nm), respectively [19].

#### 2.2.2. Synthesis of PCL–PEG co-polymer

The PCL-PEG copolymer was synthesized as described previously. In brief, 1.2 g of PCL was dissolved in dichloromethane and 0.4 mL of pyridine was added. Then, 1.2 g of 4-nitrophenyl chloroformate (NPC) was dissolved in dichloromethane and added dropwise at 0°C to the PCL solution with stirring. The solution was stirred at room temperature for 24 h under a nitrogen atmosphere to activate PCL. The resulting product (PCL-NPC) was precipitated in excess cold methanol, washed several times to remove all impurities, and dried at 45°C. Then, 0.2 g of PCL-NPC was added to a solution of 0.5 g PEG in dichloromethane and triethylamine and stirred for 24 h. The resulting copolymer was purified by excess cold methanol and dried under vacuum for at 45°C for 1 hour.

#### 2.2.3. Characterization of synthesized polymer

The PCL, PEG, and PCL–PEG copolymer were analyzed using Attenuated Total Reflectance (ATR) mode on a Perkin Elmer FT-IR spectrometer within the wavenumbers range of 450–4000 cm^-1^ [20], as well as nuclear magnetic resonance spectroscopy (^1^H-NMR, 300 MHZ; Bruker spectrometer, Germany). CDCl_3_ was used as the solvent and chemical shifts were reported in ppm (part per million) with reference to Tetramethyl Silane protons as the internal standard [21]. All experiments were performed at room temperature (25°C).

#### 2.2.4. Preparation of drug-loaded micelles

Twelve formulations of drug-loaded micelles were prepared using the emulsification solvent evaporation method with specific ratios of drug to copolymer (Table 1). To prepare the organic phase, 5 mg of MFX was dissolved in 0.5 mL methanol and added to dichloromethane solutions of copolymer (5, 10, and 25 mg co-polymer in 2 mL dichloromethane). Further 5 mg of CLM and copolymer (5, 10, and 25 mg) were dissolved in 2 mL dichloromethane. Two kinds of surfactants, polyvinyl alcohol (2%) and Tween 80 (4%), were dissolved in the aqueous phase to achieve the required hydrophilic–lipophilic balance and ensure stable emulsion. The organic phase was dispersed into 10 mL of aqueous phase at a constant rate of 0.5 mL/min using a homogenizer (Hei-TORQUE Core, Heidolph, Germany) for 10 min at 19000 rpm. The emulsions were then rotary evaporated (RV 10 auto pro V-C Complete, IKA, Germany) under reduced pressure at 45°C for 20 min to remove the dichloromethane [22].

**Table 1:** Formulations with different ratios of ingredients.

Formulation Code	MFX/co-polymer ratio	CLM/co-polymer ratio	Emulsifier
MP1	1:1	-	PVA
MP2	1:2	-	PVA
MP3	1:5	-	PVA
MT1	1:1	-	Tween 80
MT2	1:2	-	Tween 80
MT3	1:5	-	Tween 80
CP1	-	1:1	PVA
CP2	-	1:2	PVA
CP3	-	1:5	PVA
CT1	-	1:1	Tween 80
CT2	-	1:2	Tween 80
CT3	-	1:5	Tween 80

#### 2.2.5. Characterization of drug-loaded micelles

*2*.*2*.*5*.*1*. *Encapsulation efficiency (EE%) of MFX*. One mL of formulations containing MFX was centrifuged (Sigma 1–16, Germany) at 4°C for 30 min at 14,000 rpm to remove unloaded drugs. The precipitates were washed three times and dissolved in dichloromethane and methanol at a ratio of 1:10, then vortexed (VORTEX 1, IKA, Germany) to completely break up the micelles and release the drug. Incorporated drugs in the micelles were determined by HPLC using a standard curve that was plotted in the range of 10–500 μg/ml. The EE% was calculated using the following equation:

$$\text{EE}\%=\frac{\text{Mass}\;\text{of}\;\text{drug}\;\text{in}\;\text{micelles}}{\text{Mass}\;\text{of}\;\text{initial}\;\text{drug}}\;\times 100$$

*2*.*2*.*5*.*2*. *Encapsulation efficiency of CLM*. One mL of CLM-containing formulations was centrifuged at 4°C for 30 min at 14,000 rpm to precipitate the CLM-loaded micelles. The supernatant was analyzed by high-performance liquid chromatography using a reversed-phase C18 column (150 mm × 4.6 mm, 5μm) following the United States Pharmacopeia method. The mixture of methanol and 0.067 M monobasic potassium phosphate at a ratio of 13:7 (pH = 4) was used as the mobile phase at 210 nm, the flow rate was 1 mL/min and the column temperature was maintained at 50°C. The calibration curve was plotted in the range of 0.1–5 μg/ml. The EE% was calculated based on the following equations:

$$\text{EE}\%=\frac{\text{Mass}\;\text{of}\left(\text{initial}\;\text{drug}\;-\;\text{Unincorporated}\;\text{drug}\right)}{\text{Mass}\;\text{of}\;\text{initial}\;\text{drug}}\;\times 100$$

*2*.*2*.*5*.*3*. *Particle size*, *zeta potential*, *and polydispersity index*. Mean particle size, polydispersity index, and zeta potential of the micelles were measured using dynamic light scattering (ELSD-LT III, Shimadzu; Japan). The samples were appropriately diluted using deionized water and analyzed at 25°C and at a scattering angle of 90° [23].

*2*.*2*.*5*.*4*. *Transmission electron microscopy*. Transmission electron microscopy (CM-100, Philips, Netherlands) was used at 20°C to observe the morphology of the prepared micelles. One drop of micelle dispersion was deposited on a copper grid and allowed to air-dry prior to observation [24].

*2*.*2*.*5*.*5*. *In-vitro release test*. To assess the *in-vitro* release profiles of MFX and CLM from formulations MP3, MT3, CP3 and CT3, 2 mL of the micelle solution was transferred into a dialysis bag (molecular weight cutoff 13,000 Da) and immersed in 50 mL of phosphate buffer saline (pH = 7.4) containing 1% Tween 80 at room temperature with continuous stirring. At specific time intervals, 3 mL of the receiver phase was collected and replaced with 3 mL of fresh media. The amount of released drug was measured using UV–Visible spectroscopy at 410 and 230 nm for MFX and CLM, respectively [25].

#### 2.2.6. Statistical analysis

Quantitative results were expressed as mean ± standard deviation. Statistical differences were analyzed by using ANOVA (P<0.05).

## 3. Results and discussion

### 3.1. High-performance liquid chromatography (HPLC)

**Fig 1:**
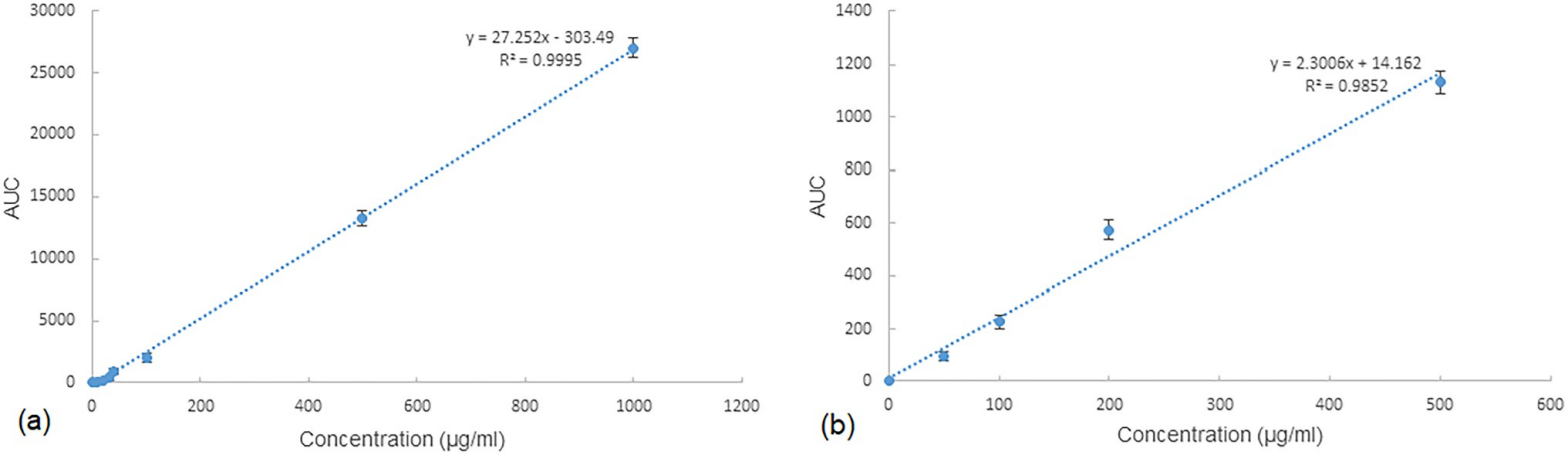
(a) Standard curve of MFX. (b) Standard curve of CLM.

Fig 1 displays the standard curves of CLM and MFX.

### 3.2. Characterization of PCL-PEG copolymer

#### 3.2.1. FTIR spectroscopy

**Fig 2:**
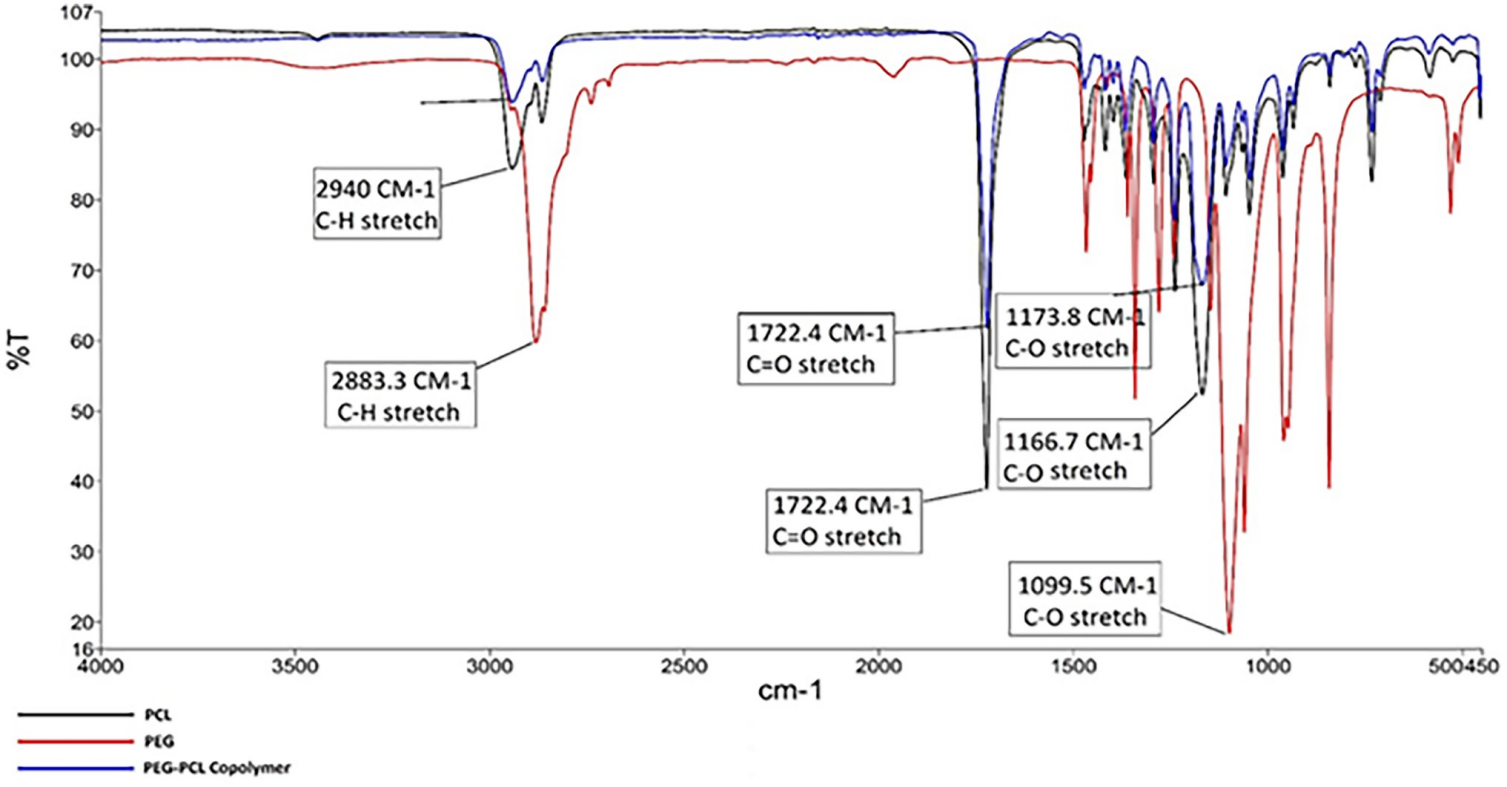
FTIR spectrum of PLC, PEG and PCL-PEG co-polymer.

Spectral analysis of PCL, PEG, and the copolymer was performed to confirm the synthesis of PCL-PEG copolymer and determine the specific and common functional groups of each polymer, as shown in Fig 2 and Table 2.

**Table 2:** Functional groups in the FTIR spectrum of PCL, PEG and PCL-PEG copolymer.

Functional groups	Wave number (cm^-1^)
Aliphatic C-H (PEG)	2883.3
C-O (PEG)	1099.5
Aliphatic C-H (PCL)	2940
C = O (PCL)	1722.4
C-O (PCL)	1166.7
Aliphatic C-H (co-polymer)	2940
C = O (co-polymer)	1722.4
C-O (co-polymer)	1173.8

#### 3.2.2. ^1^H NMR spectroscopy.

**Fig 3:**
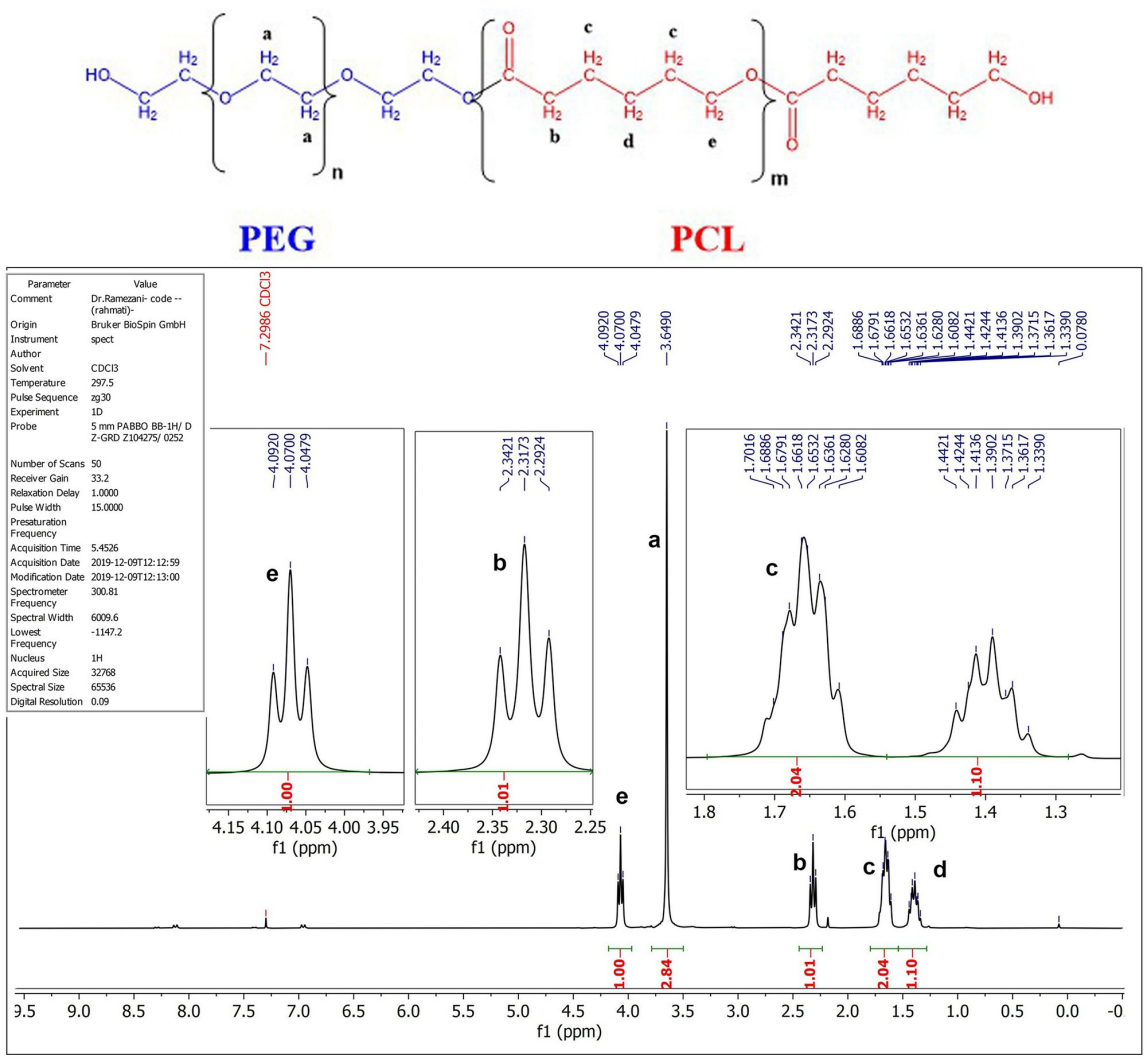
The proposed structure and ^1^H NMR spectrum analysis of synthesized PCL-PEG co-polymer. (a) CH_2_ groups of PEG, (b, c, d, e) CH_2_ groups of PCL.

The H NMR spectrum of the PCL-PEG copolymer and the spectrometer parameters are depicted in Fig 3. The hydrogens of the PEG part of the copolymer appeared as a singlet at 3.65 ppm. The PCL part of the molecules displayed four peaks in the NMR spectrum (**b-d** peaks). The most de-shielded protons (**e**, adjacent to Oxygen) appeared at 4.07 ppm as a triplet with *J = 6*.*63 Hz*. The peak at 2.32 ppm belonged to the CH2 next to the carbonyl group (**b**) and appeared as a triplet with *J = 7*.*47 Hz*. The peaks of **c** protons overlapped at about 1.67 ppm as a multiplet with an integral twice that of the other PCL peaks (**e,b,d**). The most shielded protons, based on the structure of PCL, showed a peak at approximately 1.39 ppm with a multiplet splitting pattern due to slightly different coupling constants of two adjacent methylene groups.

### 3.3. Characterization of MFX-loaded and CLM-loaded micelles

The suitability and capacity of the synthetized copolymer were assessed for loading sample hydrophilic (MFX) and lipophilic (CLM) drugs.

#### 3.3.1. Encapsulation efficiency

The EE% ranged from 41.49% to 64.57% for MFX-containing formulations and from 25.41% to 53.03% for CLM-containing formulations. The higher EE% in the MFX indicates that this hydrophilic drug can be loaded on the surface of micelles, which is a considerable area in nanoparticle drug delivery systems. On the contrary, CLM is possibly loaded in the core of micelles. The results of ANOVA showed that the drug/copolymer ratio was another factor that affected EE%, as an increase in the ratio of copolymer enhanced the capacity of micelles to load drugs and consequently increased the EE% (Table 3). Similar results were reported for Clarithromycin by Hadipour Moghaddam et al. [26]. However, ANOVA showed that different emulsifiers had no significant effect on the EE% of micelles in different formulations.

**Table 3:** Percentage of encapsulation efficacy of different formulations.

Formulation Code	EE (%) ± SE
MP1	42.95 ± 2.36
MP2	49.34 ± 4.77
MP3	64.57 ± 3.68
MT1	41.49 ± 3.29
MT2	43.04 ± 2.63
MT3	57.59 ± 4.22
CP1	35.40 ± 3.16
CP2	41.43 ± 4.08
CP3	45.10 ± 4.78
CT1	25.41 ± 2.30
CT2	37.39 ± 3.28
CT3	53.03 ± 3.86

#### 3.3.2. Particle size, zeta potential, and polydispersity index

Dynamic light scattering was used to measure the mean particle size, zeta potential, and polydispersity index of optimized formulations with drug/copolymer in the ratio of 1:5 (maximum EE%) (Table 3). The emulsifiers in the aqueous phase influenced the emulsification process and consequently the size of the micelles. The use of PVA compared to tween 80 significantly increased the particle size (Fig 3). Due to the higher viscosity of PVA compared to tween 80, the dispersion of the polymeric solution was inadequate, leading to the formation of accumulated nanoparticles after removing the organic solvent [23].

**Fig 4:**
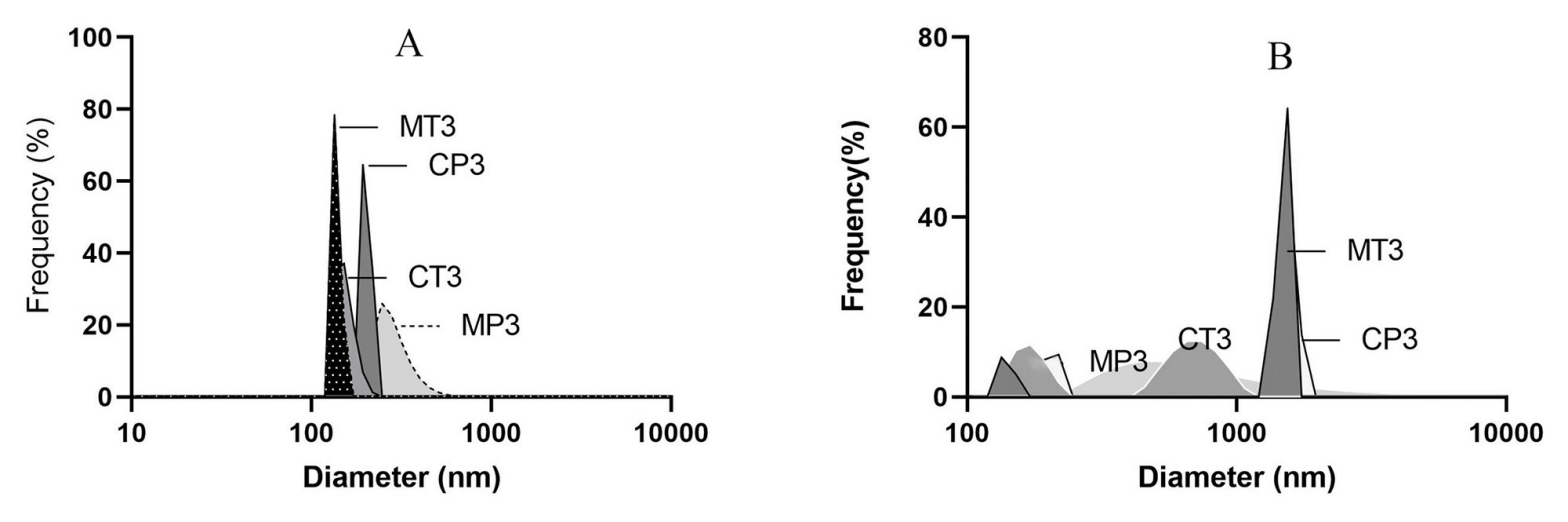
Polydispersity index graph of (a) based on intensity and (b) based on volume.

The largest particle size (272.5 ± 65.4 nm) was observed in the MP3 formulation, which had the highest EE% (64.57%) among the studied formulations. The polydispersity index of this micellar formulation was 0.418 (Fig 4). Generally, the presence of PEG on the outer layer of micelles as a shield, which reduces the zeta potential of formulations. On the other hand, cytokines adsorption was the highest by positive surface charge, followed by negative and neutral surface charge. Therefore, it seems that the simultaneous presence of the sterically large group of PEG and low negative charge reduces protein adsorption and interactions between nanomaterials and biomolecules in the surrounding environment; thus, demonstrating the stability of micellar dispersion (Table 4).

**Table 4:** Mean particle size, zeta potential and polydispersity index of optimized formulations.

Formulation code	Mean particle size (nm)	Zeta potential (mV)	Polydispersity index
MP3	272.5 ± 65.4	-6.2	0.418
MT3	129.8 ± 6.7	-5.2	0.850
CP3	190.4 ± 11.3	-6.4	0.799
CT3	144.1 ± 17.8	-3.2	0.508

#### 3.3.3. Transmission electron microscopy

**Fig 5:**
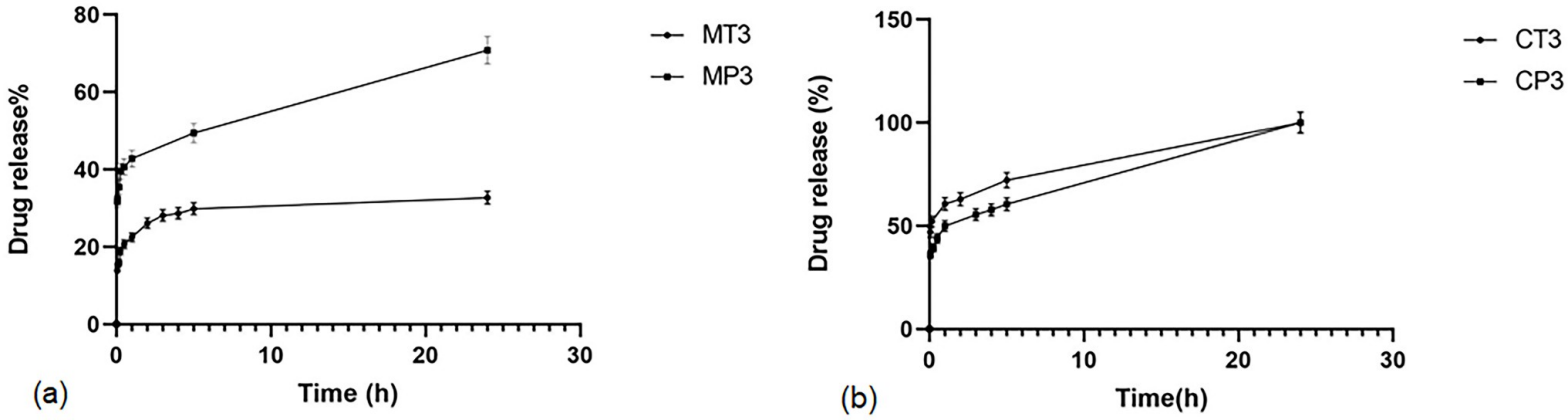
TEM images of (a) MP3 and (b) MT3 formulations.

The electron microscope images of the micelles prepared for the MP3 and MT3 formulations clearly showed the core-shell structure of the micelles, which were perfectly spherical and uniformly shaped (Fig 5).

#### 3.3.4. *In-vitro* release studies

**Fig 6:**
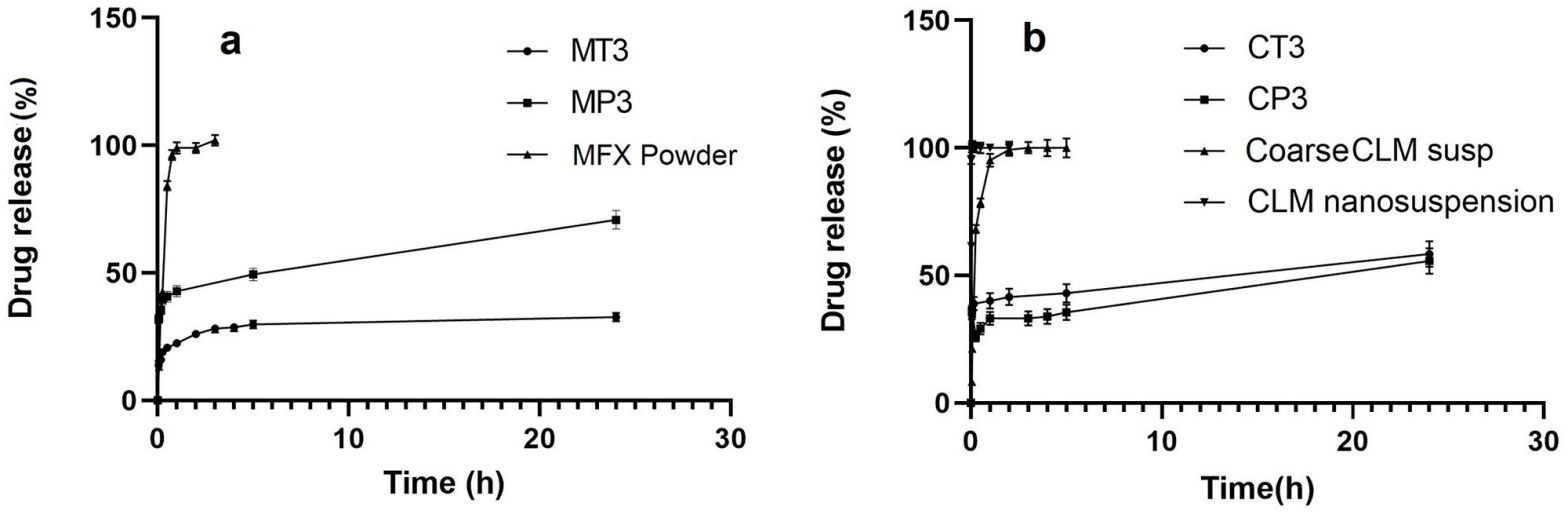
(a) Release profile of MFX from its powder, MT3 and MP3 formulations, (b) Release profile of CLM from coarse colloid, nanosuspension, CT3 and CP3 formulations.

As presented in Fig 6a, the release from MP3 and MT3 formulations occurred in two steps. During the first 120 minutes, 26.12% and 42.83% of MFX were released from MP3 and MT3, respectively. This burst release is probably due to the release portion of drug molecules that are loaded on the hydrophilic surface of the micelles. After 120 minutes, the remaining amount of the drug was slowly released up to 30% for MP3 and 70% for MT3. Regression coefficients (Table 5) showed that the release profile of MFX from both MP3 and MT3 followed the Higuchi equation within 24 hours, indicating that the release of the drug was controlled and limited by the penetration of the aqueous phase into the matrix (polymer structure of micelles). Based on the slope of the release profiles, the release rate of MT3 was slower than MP3, which was caused by the higher EE% and mean particle size of MP3 that made it take longer for the aqueous medium molecules to diffuse into micelles, dissolve drug molecules, and finally release them.

**Table 5:** Results of fitting release data to different kinetic models.

Formulation code	R^2^ for zero order	R^2^ for first order	R^2^ for Higuchi order
MT3	0.5352	0.8212	0.9734
MP3	0.327	0.3851	0.7502
CT3	0.5236	0.8042	0.9879
CP3	0.5236	0.837	0.9903

Fig 6b displays the release profiles of CP3 and CT3 formulations for CLM, which also followed a two-step release pattern and both was similar for both CT3 and CP3 formulations. During the initial 60 minutes, around 45% and 40% of CLM were released from CT3 and CP3 formulations, respectively, which may be attributed to the presence of free drugs in the solution. The regression coefficients indicated that the release of CLM from both formulations followed the Higuchi equation within 24 hours (Table 5) [27]. Additionally, the release profile of both MFX and CLM from the polymeric micelle was much slower than that of the drug powder and suspension, respectively, as reported in our other study [28].

## 4. Conclusion

According to the results of this study, polymeric micelles consisting of PEG-PCL copolymer were prepared using the emulsification solvent evaporation method, and the optimized formulations were able to load with adequate concentrations of MFX and CLM. Out of the twelve studied formulations, MT3, MP3, CT3, and CP3 had higher EE%. Their mean particle size was less than 300 nm and thus could penetrate mucous membranes well due to low surface charge. In addition, the release profiles of MFX and CLM from the polymeric micelles were in accordance with the Higuchi model.

## Supporting information

S1 Fig (TIF)
