# Supplementary figures and images for: Development and characterization of a copolymeric micelle containing soluble and insoluble model drugs
Source: PLoS One. 2023 May 25;18(5):e0286251. doi: 10.1371/journal.pone.0286251 (PMC10212155; doi:10.1371/journal.pone.0286251)

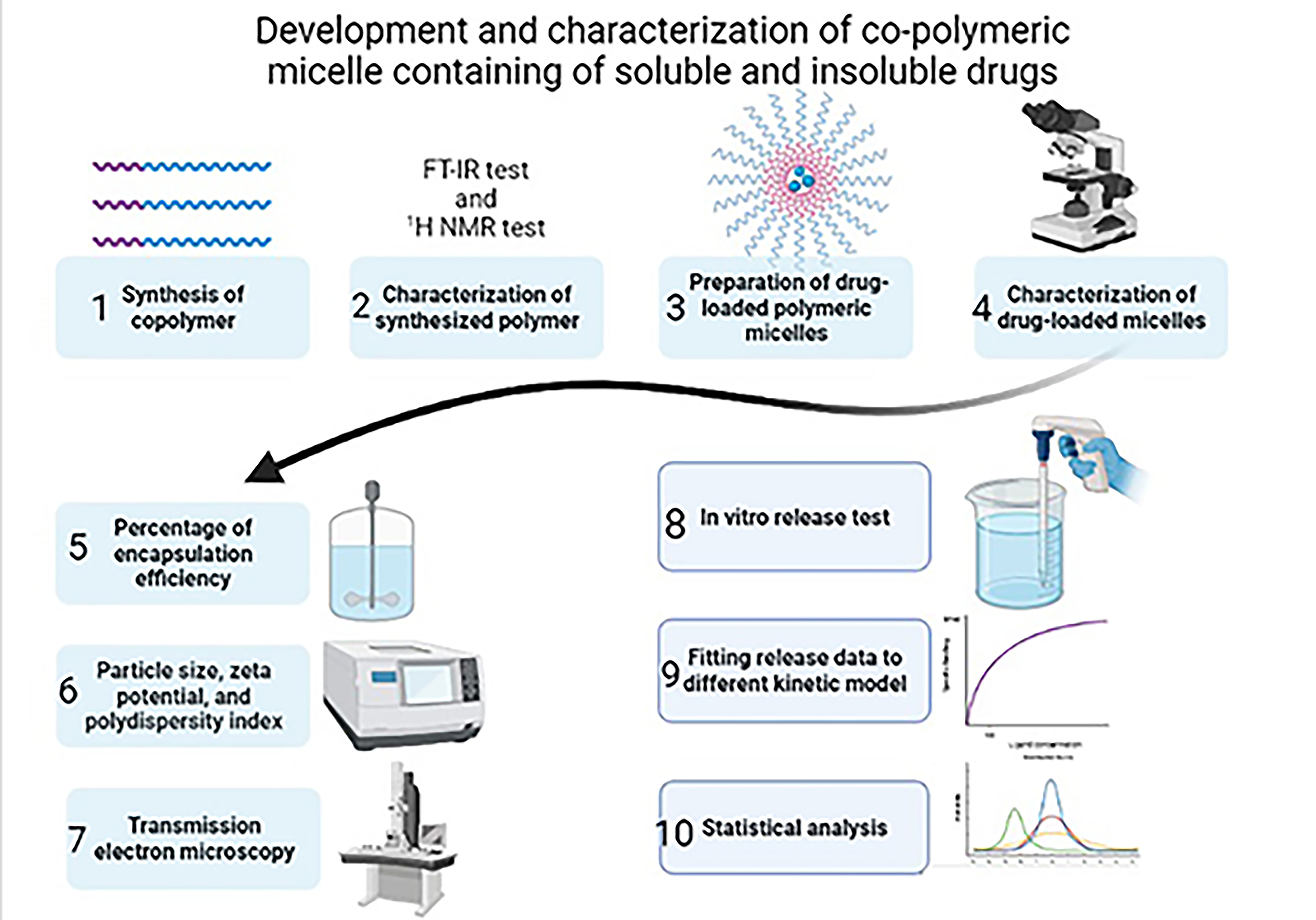

Supplement: S1 Fig — (TIF) [file pone.0286251.s001.tif]
